# Recent Advances in Diagnostics and Therapeutics for Paediatric Thyroid Cancer

**DOI:** 10.1111/jpc.70013

**Published:** 2025-02-11

**Authors:** Joel A. Vanderniet, Noemi A. Fuentes‐Bolanos, Yoon Hi Cho, David K. V. Chung, Gideon Sandler, Ali Moghimi, Bhavna Padhye, Kathy Tucker, Antoinette Anazodo, Paul Z. Benitez‐Aguirre

**Affiliations:** ^1^ Sydney Medical School, Faculty of Medicine and Health The University of Sydney Sydney New South Wales Australia; ^2^ Institute of Endocrinology and Diabetes, the Children's Hospital at Westmead Sydney New South Wales Australia; ^3^ School of Clinical Medicine, UNSW Medicine and Health University of New South Wales Sydney New South Wales Australia; ^4^ Kids Cancer Centre Sydney Children's Hospital Randwick New South Wales Australia; ^5^ Children's Cancer Institute, Lowy Cancer Centre University of New South Wales Sydney New South Wales Australia; ^6^ Department of Nuclear Medicine The Children's Hospital at Westmead Sydney New South Wales Australia; ^7^ Department of Surgery The Children's Hospital at Westmead Sydney New South Wales Australia; ^8^ Department of Surgery Westmead Hospital Sydney New South Wales Australia; ^9^ Department of Histopathology The Children's Hospital at Westmead Sydney New South Wales Australia; ^10^ Cancer Centre for Children The Children's Hospital at Westmead Sydney New South Wales Australia; ^11^ Hereditary Cancer Clinic Prince of Wales Hospital Sydney New South Wales Australia; ^12^ Nelune Cancer Centre Prince of Wales Hospital Sydney New South Wales Australia

**Keywords:** molecularly targeted therapy, multidisciplinary, ultrasound

## Abstract

**Purpose of Review:**

Paediatric thyroid cancer management traditionally relied on extrapolation from adult data and, despite good survival outcomes, often involved extensive surgical approaches and radioactive iodine (RAI) therapy with potentially life‐long complications. Increasing understanding of paediatric diagnostic techniques, molecular tumour drivers and targeted therapies will allow a more nuanced, disease‐specific comprehensive model of care. This review summarises recent developments in paediatric thyroid cancer biology, diagnosis and models of care.

**Methods:**

Review of relevant literature from the last 5 years to inform a narrative summary by a multidisciplinary team of clinician experts in paediatric thyroid cancer management.

**Findings:**

Standardised risk scoring systems will likely improve the objectivity and accuracy of paediatric thyroid nodule risk stratification on ultrasound, but further studies are needed to validate these. Identification of somatic and germline gene variants is playing a rapidly increasing role in paediatric thyroid cancer diagnosis and planning of surgical approaches and neoadjuvant and adjuvant therapies. There is growing recognition that lobectomy may achieve comparable outcomes, with reduced risk of complications, to total thyroidectomy in patients with low‐risk disease. Molecularly targeted therapies are now available for the management of advanced disease as an adjuvant, and likely neo‐adjuvant, therapy for medical debulking of large tumours and resensitisation of RAI‐resistant disease.

**Conclusions:**

The management pathways for paediatric thyroid cancer are rapidly evolving due to the increasing availability of paediatric‐specific data. As management options become more complex, interdisciplinary collaboration and shared decision‐making are ever more important.

**Summary**

- Children and adolescents with thyroid cancer should be managed in, or in conjunction with, a tertiary centre with access to a paediatric thyroid cancer multidisciplinary team meeting.
- Hemithyroidectomy may be sufficient in patients with low‐risk disease; a thorough work‐up is essential to identify appropriate patients and further studies are needed to confirm outcomes.
- Molecular analysis of the tumour plays a key role in the personalisation of paediatric thyroid cancer management and should be offered to all new and relapsed patients.
- Family history and germline screening of thyroid cancer patients will help to identify familial cancer predispositions so that individual and family follow‐up can be offered.

## 1 Introduction

Thyroid cancer is much less common in children than in adults and the incidence increases with age [1], being very rare prior to age 15 years but among the most common malignancies diagnosed in Australian adolescents aged 15–19 years. Between 1992 and 2014, Australian incidence was < 0.1 per 100 000 children under 10 years, rising to 0.33 and 1.41 per 100 000 in the age groups 10–14 and 15–19 years, respectively [2]. Females are significantly over‐represented in the adolescent age groups.

Incidence across all ages has increased in recent decades, although much of the increase has been attributed to incidental detection and early diagnosis of subclinical disease [1]. Australian Institute of Health and Welfare data show a doubling of incidence in those aged 0–19 years from 0.5 per 100 000 in 2001 to 1.0 per 100 000 in 2021 [3].

At least 95% of thyroid cancers are differentiated thyroid cancers (DTC); 90% of these are papillary carcinomas (PTC) and the remainder are follicular carcinomas (FTC). Medullary thyroid carcinoma accounts for ~4% of childhood thyroid cancers and most are associated with multiple endocrine neoplasia type 2 (MEN2) [4].

Paediatric DTC is more frequently and more widely metastatic at diagnosis than in adults yet carries very low disease‐specific mortality of < 1% [5]. Cervical lymph node metastasis is present in 60%–80% and distant metastasis in 5%–25% [6, 7]. Most paediatric DTC are sporadic, arising from acquired somatic pathogenic and likely pathogenic variants (P/LPV) in oncogenes. Less commonly, there is an identifiable risk factor, such as ionising radiation or a genetic cancer risk due to a germline P/LPV in a cancer predisposition gene [4].

Prior to the 2015 publication of the first American Thyroid Association (ATA) Guidelines for Paediatric Thyroid Nodules and Cancer, the management of paediatric thyroid nodules and cancer had mostly followed adult guidelines [8]. All children with DTC underwent total thyroidectomy followed by radioactive iodine (RAI) ablation. This approach resulted in high rates of cure, but long‐term follow‐up data had shown an increase in all‐cause mortality, mostly due to second malignancies in children treated with radiation [9].

There are important differences in molecular drivers, tumour biology, presentation and prognosis of thyroid cancer between adults and children. In recent years, the availability of paediatric‐specific molecular and clinical data has expanded significantly, allowing for the development of a more nuanced, paediatric‐specific approach [5]. A primary aim of the 2015 ATA paediatric guidelines was to provide a risk‐stratification approach and limit the use of RAI to those more likely to benefit. Other key differences to adult guidelines included a lower threshold for fine needle aspiration (FNA) of thyroid nodules and the recommendation for lobectomy rather than repeat FNA for indeterminate cytology [8].

This review will focus on further developments in the understanding of paediatric DTC since 2015 and resultant changes in management pathways, with consideration of the Australian context.

## 2 Ultrasound Evaluation of Paediatric Thyroid Nodules

Ultrasound is the first‐line radiologic modality used to assess thyroid and lymph node morphology. When assessing thyroid nodules, numerous features are considered in determining the likelihood of malignancy. Solid (or mostly solid) composition, hypoechogenicity, taller‐than‐wide shape, irregular margins, extra‐thyroidal extension, increased intranodular blood flow, calcification and enlarged regional lymph nodes all confer suspicion for malignancy [10]. No single feature is sufficient to differentiate malignant from benign nodules. In adults, multiple scoring systems have been validated to integrate these features and provide a recommendation on whether to proceed to FNA. These include the ATA adult risk stratification method [11] and several versions of the Thyroid Image Reporting and Data System (TI‐RADS) [12], initially developed by the American College of Radiology (ACR). In contrast, the ATA paediatric guidelines recommend FNA in all solid or partially cystic nodules ≥ 1 cm or nodules of any size if any suspicious sonographic features are present [8].

More recently, several studies have sought to assess the validity of ultrasound‐based thyroid nodule scoring systems in paediatrics. Results have been mixed, with some recommending against their use due to inadequate sensitivity [13, 14], whereas others concluded that they perform adequately and comparatively to their use in adults [15, 16, 17, 18]. However, most authors agree that nodule size criteria need to be modified or disregarded in children due to the higher incidence of malignancy in smaller nodules. In addition, clinicians should be aware that the rarer diffuse sclerosing variant PTC is more commonly seen in younger patients and appears sonographically as non‐nodular, diffuse microcalcifications throughout the thyroid [19, 20].

Recent studies in adult cohorts have examined the value of ultrasound elastography and microvascular flow imaging in risk‐stratifying thyroid nodules. Some have reported a modest increase in diagnostic performance when used in combination with conventional ultrasound scoring systems [21, 22, 23]. Further research is required before they can be recommended for routine use and in paediatrics [24].

There is increasing interest in the use of artificial intelligence in the sonographic analysis of thyroid nodules. Several groups have developed deep learning algorithms based on adult thyroid nodule images [25, 26, 27]. Yang et al. evaluated one of these algorithms in a cohort of 139 children and young adults and found it had sensitivity comparable to ACR TI‐RADS and higher than radiologists' impressions, but specificity lower than both [28]. Further work is needed to train deep learning algorithms for paediatric use, but large image banks are needed and the rarity of paediatric thyroid cancer poses a barrier to rapid progress.

## 3 Interpretation of Cytopathology

The characterisation of thyroid nodules suspicious for malignancy relies on FNA and cytopathology, reported using the Bethesda System for Reporting Thyroid Cytopathology [29]. This consists of 6 categories, with a corresponding risk in adults of DTC from 2% to 7% in the benign category up to 97%–100% in the malignant category. Thyroid nodules in children are more likely to be malignant than in adults and the risk of malignancy for each Bethesda category is also higher. This is reflected in the 2023 revision, which includes separate paediatric risk of malignancy estimates for each category (Table 1) [29].

**TABLE 1:** 2023 Bethesda system for reporting thyroid cytopathology in paediatric patients with implied risk of malignancy (ROM).

Diagnostic category		ROM mean (range)
I	Nondiagnostic	14% (0–33)
II	Benign^a^	6% (0–27)
III	Atypia of undetermined significance	28% (11–54)
IV	Follicular neoplasm^b^	50% (28–100)
V	Suspicious for malignancy	81% (40–100)
VI	Malignant	98% (86–100)

*Note*: Adapted, with permission, from Ali et al. [29].

^a^ ROM is skewed by selection bias because most thyroid nodules classified as benign do not undergo surgical excision.

^b^ Includes cases of follicular neoplasm with oncocytic features (formerly Hurthle cell neoplasm).

Approximately 35% of paediatric thyroid nodule FNAs return an indeterminate result (Bethesda III or IV) [8]. The ATA paediatric guidelines recommended lobectomy rather than repeat FNA, due to evidence that 28% and 58%, respectively, of these nodules are malignant [8]. The more recent European Thyroid Association paediatric guidelines suggest that repeat FNA after 6 months is reasonable, proceeding to lobectomy if repeated indeterminate cytology [30]. Both guidelines recommend repeat FNA after 3–6 months for nondiagnostic cytology [8, 30]. Nodules with benign cytology warrant repeat ultrasound in 6–12 months, then reducing frequency if stable or repeat FNA if increasing in size [8, 10, 30].

## 4 Genetic Testing for Somatic Variants

Molecular testing of FNA samples to help determine the likelihood of malignancy in indeterminate nodules is becoming more common in adults. Commercially available gene panels have demonstrated positive predictive values of 66% and negative predictive values of up to 95% for malignancy in Bethesda III and IV nodules [31]. A negative result can therefore be of significant value in surgical planning. In addition, certain molecular features are associated with a higher risk of invasive disease and may inform planning for a more extensive surgical approach, reducing the need for repeat operations [32].

**FIGURE 1:**
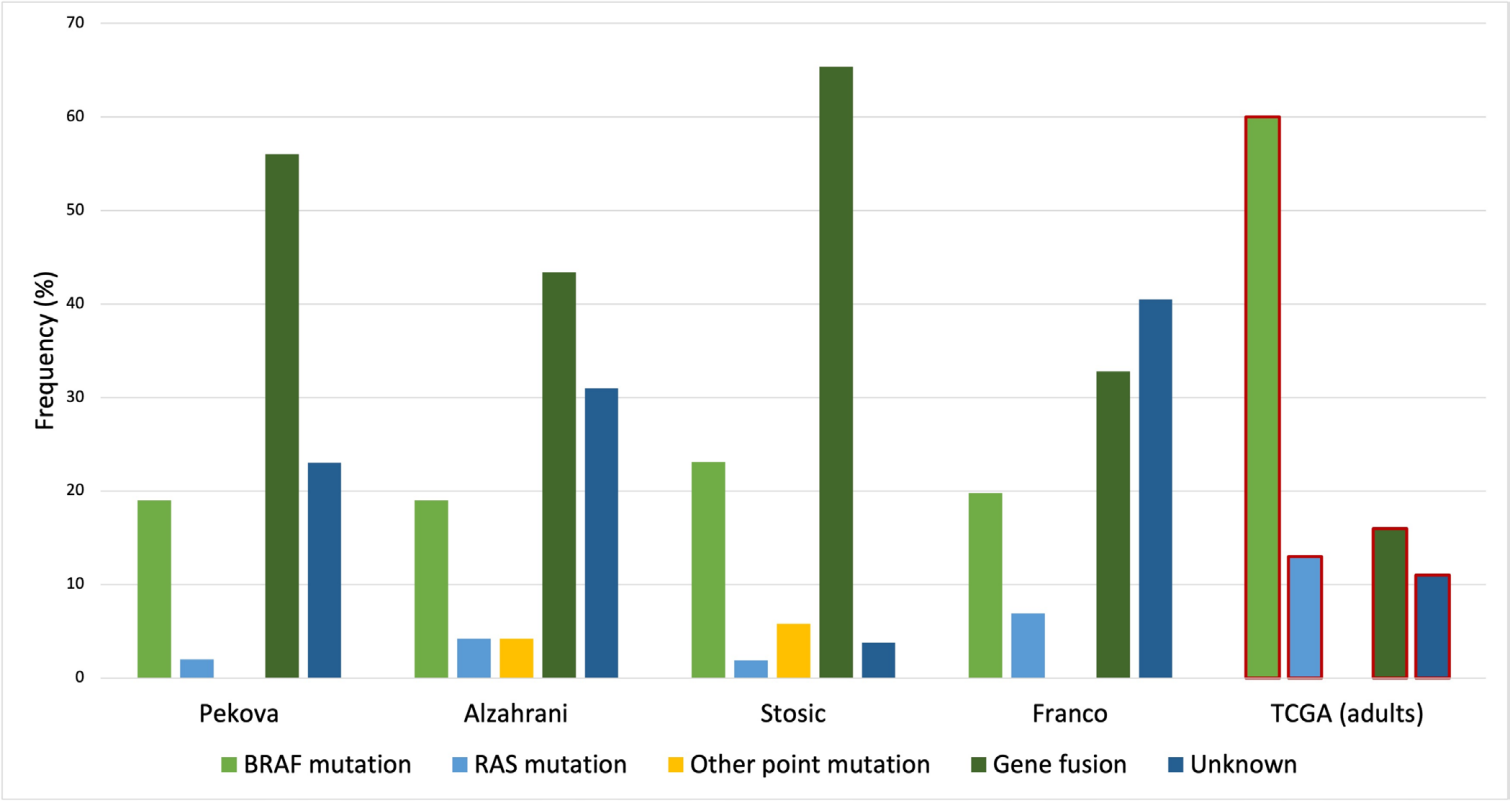
Frequency of point mutations and gene fusions in four paediatric studies compared with adult data from The Cancer Genome Atlas (TCGA). Note differing methodologies between studies. Citations: Pekova et al. [35], Alzahrani et al. [36], Stosic et al. [37], Franco et al. [38] and TCGA [39].

In paediatric studies, *RET*, *NTRK* and *ALK* fusions and *BRAF* point mutations are associated with malignancy in almost all cases [33, 34]. In contrast to adults, gene fusions are found more commonly than point mutations (Figure 1) [35, 36, 37, 38, 39]. *BRAF* mutations are less common than in adults, and their prevalence appears to increase with age [38, 40]. In a recent study, *BRAF* mutations were identified in only 20% of paediatric DTC patients [38], compared with 60% of adults in The Cancer Genome Atlas data [39]. Only 1 of 11 patients under age 10 years had a *BRAF* mutation; the remaining 10 had a gene fusion [38]. *RAS*, *DICER1* and *PTEN* variants can be associated with a range of paediatric thyroid diseases, from low‐risk PTC and adenomas to aggressive and poorly differentiated cancers [33, 34]. Table 2 presents the common somatic driver variants seen in paediatric thyroid cancer.

**TABLE 2:** Somatic driver variants are seen in paediatric thyroid cancer and their corresponding typical histology, invasive potential and molecularly targeted therapies.

Variant	Histology	Invasive potential	Incidence in paediatric DTC	Molecularly targeted therapy
*RET* fusion or point mutation	PTC MTC	High Depends on *RET* codon	26%–30%	Selpercatinib Pralsetinib
*NTRK* fusion	PTC	High	7%–18%	Larotrectinib Entrectinib
*ALK* fusion	PTC MTC	High Limited data	2%–6%	Crizotinib Ceritinib Alectinib Ensartinib
*BRAF* ^*V600E*^ point mutation	PTC	Intermediate	19%–23%	Dabrafenib with tremetinib Vemurafenib
*RAS* point mutation *PTEN* point mutation *DICER1* point mutation *PAX8/PPAR𝛾* fusion	PTC, FTC, benign FTC	Low	2%–7% 0%–3% 0%–5% 0%–2%	Multikinase inhibitors only: Lenvatinib Sorafenib
*TSHR* point mutation *GNAS* point mutation	Benign	N/A	N/A	N/A

*Note*: Incidence data from Pekova et al. [35], Alzahrani et al. [36], Stosic et al. [37] and Franco et al. [38]. Adapted, with permission, from Bauer [4].

Abbreviations: DTC, differentiated thyroid cancer; FTC, follicular thyroid carcinoma; MTC, medullary thyroid carcinoma; PTC, papillary thyroid carcinoma.

Whilst paediatric studies using a gene panel approach performed in FNA samples have demonstrated high specificity, they had high false‐negative rates and therefore cannot currently be recommended as a standard method to rule out malignancy [33]. It is expected that the addition of microRNA testing to gene variant analysis will increase the overall sensitivity to around 70%, but still not sufficient to confidently exclude malignancy [41].

The finding of a *RET*, *NTRK* or *ALK* fusion indicates a high risk for invasive disease and warrants a total thyroidectomy with central ± lateral neck dissection, while most point mutations are associated with a lower invasive potential and may allow for a less extensive surgical approach, depending on ultrasound and cytology findings [4, 34]. However, further research is required before routine molecular testing of FNA samples from paediatric thyroid nodules can be recommended [42].

Post‐operative testing of surgical specimens for molecular analysis using immunohistochemistry and fluorescence in situ hybridisation (FISH) is now routine practice in some centres [43]. Molecular information at this stage helps to characterise the invasiveness of the tumour and informs adjuvant therapy and ongoing surveillance. The presence of a *RET*, *NTRK* or *ALK* fusion is associated with an increased risk of lymph node and pulmonary metastasis and may even predict invasive disease better than histology [40, 44, 45]. Identification of a molecular tumour driver also provides the opportunity for the use of molecularly targeted therapies for advanced or RAI‐resistant disease.

In Australia, Medicare Benefits Schedule funding is now available for molecular testing of locally advanced or metastatic paediatric thyroid cancer tissue with suspected *NTRK* fusions, giving access to NTRK inhibitors if positive [46]. Such programs, linking molecular analysis with drug access, will be key to guiding future models of care.

## 5 Genetic Testing for Cancer Predisposition Conditions

Genetic predisposition is the second most common identifiable risk factor for the development of thyroid cancer [4]. Some cancer predisposition syndromes are associated with an increased risk of thyroid cancer in addition to other non‐thyroidal cancers (Table 3) [30, 47]. Therefore, the identification of a germline P/LPV in a cancer predisposition gene enables ongoing surveillance and early detection of further cancers, as well as cascade testing to identify family members at risk.

**TABLE 3:** Cancer predisposition syndromes associated with thyroid nodules and cancer.

Syndrome	Gene and mode of inheritance	Type of thyroid neoplasia	Other clinical features	Other tumours
Familial adenomatous polyposis	*APC* Autosomal dominant (20% de novo)	PTC (cribriform‐morular variant)	Absence and delayed eruption of teeth	Hepatoblastoma Medulloblastoma GI tract polyps
Carney complex	*PRKAR1A* Autosomal dominant (30% de novo)	Multinodular goitre Follicular adenoma DTC	Brown/black lentigines of skin, lips, oral mucosa Epithelioid‐type blue naevi	Benign adrenal tumours Pituitary tumours Sertoli cell tumours Breast ductal adenoma Osteochondromyxoma Schwannoma
DICER1 syndrome	*DICER1* Autosomal dominant	Multinodular goitre DTC (second‐hit somatic mutation in *DICER1*) Poorly differentiated carcinoma	Macrocephaly	Pleuropulmonary blastoma Ovarian tumours Cystic nephroma Ciliary body medulloepithelioma Embryonal rhabdomyosarcoma Nasal hamartoma Pituitary blastoma Pineoblastoma Wilms tumour Juvenile intestinal hamartomas
PTEN hamartoma tumour syndrome	*PTEN* Autosomal dominant (> 10% de novo)	Multinodular goitre Follicular adenoma DTC (especially FTC)	Macrocephaly Dolichocephaly Autism, developmental delay Oral papillomas Facial papules Keratoses	Benign and malignant tumours of Breast Colon Endometrium Kidney Lipomas, haemangiomas Cerebellar dysplastic gangliocytoma
Werner syndrome	*WRN* Autosomal recessive	DTC Anaplastic thyroid cancer	Short stature Cataracts Multiple skin abnormalities Hypogonadism Osteoporosis Premature atherosclerosis Diabetes mellitus	Malignant melanoma Meningioma Soft tissue sarcomas Leukaemia Bone tumours
MEN2A	*RET* Autosomal dominant	MTC	Hirschsprung disease Cutaneous lichen amyloidosis	Phaeochromocytoma Parathyroid adenoma/hyperplasia
MEN2B	*RET* Autosomal dominant	MTC	Alacrima in infancy Infantile feeding difficulties, constipation and hypotonia Distinctive facies, enlarged lips Joint laxity, SCFE, pes cavus Marfanoid body habitus	Phaeochromocytoma Mucosal neuromas Intestinal ganglioneuromatosis

*Note*: Adapted, with permission, from Lebbink et al. [30].

Abbreviations: DTC, differentiated thyroid carcinoma; FTC, follicular thyroid carcinoma; MEN, multiple endocrine neoplasia; MTC, medullary thyroid carcinoma; PTC, papillary thyroid carcinoma; SCFE, slipped capital femoral epiphysis.

Familial non‐medullary thyroid cancer is a term used for DTC occurring in 2 or more first‐degree relatives in an autosomal dominant inheritance pattern, without the other clinical features or increased risk of non‐thyroidal tumours typical of cancer predisposition syndromes [4]. It tends to present at a younger age than sporadic DTC and shows clinical anticipation between generations. A single germline locus has not been identified as the aetiology of this entity, but some authors have attributed heritability, at least in part, to a variety of genes, suggesting that polygenic risk might play a role in the underlying cause of this condition [48].

Outside the research setting, germline testing is currently advised for thyroid cancer patients with clinical features or family history suggestive of a cancer predisposition syndrome, or when a *DICER1* or *PTEN* P/LPV has been identified on somatic testing. The Dutch Paediatric Thyroid Cancer Consortium recently suggested that germline testing may be warranted in all paediatric thyroid cancer patients, based on their identification of a germline variant in 13% of 81 paediatric thyroid cancer patients, only 50% of whom had clinical features suggestive of a cancer predisposition syndrome [48]. The Cancer Institute NSW eviQ guidelines recommend for all tumour types that genetic testing is indicated when the probability of identifying a clinically actionable germline P/LPV in a cancer or tumour predisposition gene is ≥ 10% [49]. Both somatic and germline testing are currently available to all paediatric thyroid cancer patients in Australia through the Zero Childhood Cancer Program [50].

All children and adolescents with medullary thyroid cancer should undergo germline testing as nearly all cases are associated with MEN2 [4].

## 6 Surgical Approach

Due to the common multifocality of paediatric PTC [51, 52, 53, 54], the ATA and ETA paediatric guidelines recommended total or near‐total thyroidectomy for the majority of children with PTC [8, 30]. Long‐term follow‐up of paediatric patients with PTC had shown a 6% incidence of local recurrence after total thyroidectomy, compared with 35% for lobectomy [9]. Total thyroidectomy also facilitates the use of radioactive iodine for the detection and treatment of residual disease and serum thyroglobulin measurement for long‐term surveillance. Both guidelines recommended bilateral central neck dissection if any evidence of extrathyroidal extension or metastasis pre‐ or intra‐operatively, with lateral neck dissection reserved for proven lateral lymph node metastasis.

In contrast, adult DTC management has seen a shift toward less extensive surgery for low‐risk cancers, based on studies showing a lower risk of surgical complications without an increased risk of recurrence or mortality compared with total thyroidectomy [11]. Given the higher risk of surgical complications and low disease‐specific mortality in children with DTC, there is a need to determine whether there is a subset of paediatric patients who could be treated with lobectomy rather than total thyroidectomy [5].

Recent studies have shown rates of bilateral disease of only 5%–16% in children with unifocal PTC and no clinical lymph node metastases, compared with 53%–65% in those with multifocal tumours [53, 55]. In the single‐centre study by Sugino et al., in patients with no clinical lymph node metastases or extrathyroidal tumour extension, lobectomy resulted in equivalent 20‐year disease‐free survival to total thyroidectomy [56]. A propensity‐matched survival study using US cancer registries showed that lobectomy and total thyroidectomy resulted in equivalent 10‐year overall and disease‐specific survival in children with low‐risk thyroid cancer without nodal or distant metastases, although it did not evaluate associations with disease recurrence [57].

There is growing evidence that some children with low‐risk thyroid cancer may achieve good outcomes with less extensive (and lower‐risk) initial surgery. This group may include those without clinical evidence of lymph node metastases or multifocality and without ultrasound features or genetic findings associated with invasive disease. Regardless of approach, surgery should be performed by an experienced thyroid surgeon in a tertiary centre with access to a multidisciplinary team to support decision‐making [8, 30, 42].

## 7 Molecularly Targeted Therapies

Despite metastasis being more common and widespread at presentation in paediatric DTC than in adults, disease‐specific mortality is low. RAI is a highly effective, targeted therapy and the current standard of care for persistent post‐surgical disease [4]. Nevertheless, < 20% of paediatric patients with pulmonary metastasis treated with RAI achieve complete remission, and this metastatic disease becomes a chronic condition [58, 59, 60, 61]. While most patients will see a reduction in disease burden, some will be RAI‐resistant. As molecular analysis of thyroid cancer becomes more commonplace, the opportunity for molecularly targeted therapy will become available to more patients with advanced and RAI‐resistant disease.

Multi‐kinase inhibitors were the first small molecule inhibitors available for this purpose. Molecularly targeted drugs are now available for *NTRK*, *RET* and *ALK* fusions and *BRAF* variants (Table 2). NTRK and RET inhibitors have shown efficacy of 80%–90% to cause regression of disease and to increase RAI‐avidity in adults, and a smaller number of children, with advanced disease [40, 62, 63, 64]. Long‐term use to control metastatic disease has generally been well‐tolerated, although side effects are a limiting factor in some patients. Larotrectinib is now funded through the Pharmaceutical Benefits Scheme in Australia for patients with metastatic or unresectable locally advanced *NTRK* fusion‐positive disease.

The use of these agents for RAI resensitisation in RAI‐refractory disease is a promising alternative strategy and is currently being studied in paediatric cohorts (ClinicalTrials.gov ID NCT05024929). Molecularly targeted inhibitors have greatly increased RAI uptake in some patients, necessitating lower RAI doses to prevent pulmonary fibrosis and undue risk of secondary malignancy [40, 64]. The optimal dosing method in this circumstance has not been established, but dosimetry has been used by some to optimise the balance between efficacy in target tissues and limiting radiation exposure to sensitive tissues. The blood/whole body method of dosimetry requires daily blood tests over 3–5 days to determine radioactivity ‘as‐high‐as‐safely administrable’. Lesion‐based dosimetry may be used for macroscopic, measurable lesions, allowing the calculation of a specific absorbed dose for the lesion [65, 66].

A third potential indication is neoadjuvant molecularly targeted therapy debulking of tumours with high surgical risk for anatomical reasons. This approach has achieved a sufficient reduction of tumour size to allow successful resection of previously inoperable tumours [67].

Further research is required to establish optimal strategies for the use of molecularly targeted therapies in children, including treatment duration. As more experience is gained and their risk profile becomes well understood, they will likely find use in a growing proportion of paediatric thyroid cancer patients to increase rates of remission following primary surgery and RAI, rather than being limited to patients with advanced disease.

## 8 Conclusions

Much has changed since the publication of the ATA paediatric thyroid cancer guidelines in 2015. According to that paradigm, most children with a thyroid nodule with any suspicious sonographic features would proceed to FNA and all those without clearly benign cytology would proceed to surgery—total thyroidectomy if cytology showed malignancy. Children with demonstrated metastases would then undergo RAI treatment, multiple times if their disease persisted, and yet some would have persistent distant metastatic disease long‐term.

The paradigm ahead of us is more nuanced and targeted to the specifics of each patient's disease. Ultrasound risk stratification systems will be validated for paediatric use and further refined, reducing unnecessary invasive procedures. Pre‐surgical genetic analysis will more accurately predict tumour behaviour and influence the extent of surgery performed and other treatments offered. Patients with low‐risk disease may avoid total thyroidectomy, reducing their risk of surgical complications and avoiding the need for lifelong thyroid hormone replacement therapy. Molecularly targeted therapies will provide another avenue for the treatment of advanced and RAI‐resistant disease and improve the efficacy of primary treatments.

As management pathways become more nuanced, interdisciplinary collaboration and shared decision‐making at each stage becomes fundamental. As the platforms for these patient discussions, regular multidisciplinary meetings are now held in most states of Australia and New Zealand, and their expertise is available to any clinician managing a child with thyroid cancer in these countries. Discussion of cases in a recognised multidisciplinary meeting is now part of the standard of care for paediatric thyroid cancer [8, 10, 30, 42] as we strive to continually improve outcomes and minimise risks for this group of patients.

## Conflicts of Interest

The authors declare no conflicts of interest.
